# Obstetric training in Emergency Medicine: a needs assessment

**DOI:** 10.3402/meo.v21.28930

**Published:** 2016-06-28

**Authors:** Adam James Janicki, Courteney MacKuen, Alisse Hauspurg, Jamieson Cohn

**Affiliations:** 1 Department of Emergency Medicine, Alpert Medical School of Brown University, Providence, RI, USA; 2 Department of Obstetrics and Gynecology, Alpert Medical School of Brown University, Women & Infants Hospital, Providence, RI, USA

**Keywords:** Emergency Medicine, obstetrics, graduate medical education, curriculum development

## Abstract

**Background:**

Identification and management of obstetric emergencies is essential in emergency medicine (EM), but exposure to pregnant patients during EM residency training is frequently limited. To date, there is little data describing effective ways to teach residents this material. Current guidelines require completion of 2 weeks of obstetrics or 10 vaginal deliveries, but it is unclear whether this instills competency.

**Methods:**

We created a 15-item survey evaluating resident confidence and knowledge related to obstetric emergencies. To assess confidence, we asked residents about their exposure and comfort level regarding obstetric emergencies and eight common presentations and procedures. We assessed knowledge via multiple-choice questions addressing common obstetric presentations, pelvic ultrasound image, and cardiotocography interpretation. The survey was distributed to residency programs utilizing the Council of Emergency Medicine Residency Directors (CORD) listserv.

**Results:**

The survey was completed by 212 residents, representing 55 of 204 (27%) programs belonging to CORD and 11.2% of 1,896 eligible residents. Fifty-six percent felt they had adequate exposure to obstetric emergencies. The overall comfort level was 2.99 (1–5 scale) and comfort levels of specific presentations and procedures ranged from 2.58 to 3.97; all increased moderately with postgraduate year (PGY) level. Mean overall percentage of items answered correctly on the multiple-choice questions was 58% with no statistical difference by PGY level. Performance on individual questions did not differ by PGY level.

**Conclusions:**

The identification and management of obstetric emergencies is the cornerstone of EM. We found preliminary evidence of a concerning lack of resident comfort regarding obstetric conditions and knowledge deficits on core obstetrics topics. EM residents may benefit from educational interventions to increase exposure to these topics.

The identification and management of obstetric and gynecologic (Ob/Gyn) emergencies is an essential skill in emergency medicine (EM). Obstetric emergencies characteristically occur infrequently but are a major cause of maternal and fetal mortality (1). Treatment decisions must be made without hesitation, and there are potentially life-threatening consequences for incorrect management decisions (2). Nationally, there are over 750,000 annual emergency department (ED) visits for gynecologic and obstetric complaints (3). Experience with Ob/Gyn patients is therefore an important part of EM, but there is often inadequate exposure to pregnant patients during EM residency training. This is especially true in academic hospitals with dedicated obstetric triage areas and Ob/Gyn residents.

To date, there is little data describing effective ways to teach EM residents this material, and no standardized obstetrics curriculum for EM residents exists. The Accreditation Council for Graduate Medical Education (ACGME) requires a half-month of obstetric experience or completion of 10 vaginal deliveries, but it is unclear whether this provides the experience necessary to become competent (4). Many residency programs have one dedicated Ob/Gyn rotation, often during the intern year. This may establish a foundation of knowledge, but exposure to complications of pregnancy is subject to chance and the lack of sustained exposure may be a detriment to resident comfort with obstetric emergencies.

Curriculum development in medical education begins with the identification of a problem with a general and then targeted needs assessment (5). In order to identify a curriculum for obstetric training in EM that would ensure clinical competency required for emergency practice, we performed a needs assessment to establish residents’ attitudes, beliefs, and knowledge of basic obstetric emergency topics. The study was conceptualized as a preliminary investigation to inform hypotheses for further research regarding the potential need for and effect of educational interventions.

## Methods

We created a 15-item survey designed to elicit resident attitudes and knowledge related to obstetric emergencies. Attitudes and beliefs were addressed with questions regarding residents’ exposure to obstetric emergencies and their comfort regarding eight common presentations and skills: first, second, and third trimester bleeding, decreased fetal movement, labor, cervical checks, pelvic ultrasound, and lower abdominal pain in pregnancy – conditions deemed either ‘critical’ or ‘emergent’ by the 2013 EM Model Curriculum (6). Overall comfort level was determined by a composite score of all subjective items. Knowledge of obstetric emergencies and procedures was assessed through five multiple-choice questions regarding the management of preterm labor, second-trimester bleeding, normal vaginal delivery, and the interpretation of pelvic ultrasound and cardiotocography images. All subjective items were answered with a 5-point scale with anchors of 1-‘not comfortable’, 3-‘somewhat comfortable’, and 5-‘completely comfortable’. All multiple-choice questions had five answer choices; residents were asked to select the most correct choice.

Multiple-choice questions were adapted from standardized study questions for the National Board of Medical Examiners Ob/Gyn medical student shelf examination; these are designed to target third-year medical students. Questions were piloted among EM and Ob/Gyn faculty and residents to ascertain face validity and were revised based on feedback. Difficulty indexes were calculated for each multiple-choice question.

The survey was administered online via SurveyMonkey in September 2014, with the link distributed to program directors across the United States utilizing the Council of Emergency Medicine Residency Directors (CORD) listserv. Program directors were asked to distribute the study invitation and survey link to their residents via email. There were no incentives for participation.

Given the presence of both 3- and 4-year residency programs, postgraduate year (PGY) 3 and 4 results were combined in analysis, similar to the reporting of the American Board of Emergency Medicine In-Service Examination scores. Subjective item scores, composite scores of all subjective items, and mean composite scores on the multiple-choice questions were calculated and scores for each PGY level were compared using one-way ANOVA tests. Individual multiple-choice question scores were also calculated, stratified by PGY level and compared using chi-square tests. All means are reported with standard deviations (SD). A *p*-value less than 0.05 was considered statistically significant.

All study procedures were approved by the Rhode Island Hospital institutional review board.

## Results

The survey was completed by 212 residents from 28 different states, representing 55 of 204 (27%) accredited ED residency training programs belonging to CORD, and 11.2% of the 1,896 eligible residents. Participating residents stratified by PGY level and geographical region are presented in Table 1.

**Table 1:** Respondent characteristics

	No. of respondents (%)
PGY level	
PGY1	47 (22.2)
PGY2	78 (36.8)
PGY3/4	87 (41.0)
Location	
Northeast^a^	87 (41)
South^b^	61 (28.8)
Midwest^c^	38 (17.9)
West^d^	25 (11.8)

a Northeast: Maine, Massachusetts, Rhode Island, Connecticut, New York, New Jersey, Pennsylvania.

b South: Delaware, West Virginia, North Carolina, Kentucky, Tennessee, Georgia, Florida, Mississippi, Arkansas, Louisiana, Texas.

c Midwest: Ohio, Illinois, Michigan, Minnesota, Missouri.

d West: Colorado, Arizona, Utah, California, Washington.

Fifty -six percent of residents felt that they had adequate exposure to obstetric emergencies. The composite score for comfort level of managing obstetric emergencies for all respondents was low, 2.99 (SD 0.70), increasing modestly with advancing PGY level (PGY1=2.54, SD 0.66, PGY2=2.99, SD 0.60, PGY3/4=3.24, SD 0.69; *p*<0.001). The comfort levels of the eight specific presentations and procedures ranged from 2.58 (SD 1.04) to 3.97 (SD 0.94) and generally increased with PGY level (Table 2).

**Table 2:** Subjective measurements

	Mean comfort level (1–5)				
		
Presentation/procedure	All residents	PGY 1	PGY 2	PGY 3/4	*p*
Composite score	2.99	2.54	2.99	3.24	<0.001*
1st Trimester bleeding	3.97	3.27	3.94	4.37	<0.001*
2nd Trimester bleeding	3.2	2.57	3.21	3.53	<0.001*
3rd Trimester bleeding	2.72	2.36	2.69	2.93	0.01*
Decreased fetal movement	2.6	2.3	2.56	2.8	0.01*
Labor	2.95	2.68	3.12	2.95	0.054
Cervical checks	2.58	2.26	2.46	2.87	0.002*
Pelvic ultrasound	2.65	2.26	2.64	2.86	0.02*
Lower abdominal/pelvic pain in pregnancy	3.26	2.62	3.28	3.6	<0.001*

* Values are statistically significant (*p<*0.05).

Mean percent correct across all multiple-choice questions was 58.0% (SD 20.9%) with no statistical difference by PGY level (PGY1=56.2%, SD 21.1%; PGY2=59.7%, SD 22.0%; PGY3/4=57.5%, SD 20.0%; *p*=0.622). Difficulty indexes for each question by topic included: preterm labor 0.49, cardiotocography 0.41, second-trimester bleeding 0.46, normal vaginal delivery 0.90, and pelvic ultrasound 0.62. There was no statistical difference by PGY level on individual question performance.

## Discussion

Identification and management of obstetric emergencies is essential in EM. In fact, the Emergency Medical Treatment and Labor Act, which requires all patients presenting at ED be evaluated and stabilized, specifically mandates that all pregnant patients in presumed labor be evaluated by a physician (7). Obstetric emergencies characteristically occur infrequently, making it difficult to establish and maintain competency. Further, obstetric emergencies are often coupled with high risks; therefore, it is imperative that emergency physicians are competent in caring for these patients. To date, no needs assessment has been performed regarding obstetric training in EM to inform the improvement of this component of residency education.

Currently, ACGME requirements are relatively limited and quite broad (4). Given that only about half of the residents felt that they had enough experience with these patients, the limited time currently required by the ACGME may not allow for adequate exposure to obstetric emergencies. Of note, in our sample, even residents at the end of their training did not feel ‘somewhat comfortable’ (response less than three) managing over 50% of the surveyed topics.

Although half of the residents surveyed felt they had adequate obstetric training, overall knowledge scores were low. Thus, EM residents may be unaware of their educational deficits; therefore, it was important to evaluate resident knowledge regarding managing these patients. The average composite score on the multiple-choice questions was only 58%, suggesting that current requirements may be insufficient in training knowledgeable resident physicians. Even more concerning is that variation in testing performance was not attributable to training level – all residents in our sample demonstrated educational deficits on core obstetric topics.

The ACGME requirement of completion of 10 vaginal deliveries for all EM residents potentially homogenizes EM education regarding this subject matter. Based on our testing, this standardization is associated with improved competency, given the difficulty index of 0.90 for the question asking to identify the correct steps of a normal vaginal delivery. The lower difficulty indexes on the remaining multiple-choice questions suggest that consideration should be given to the potential benefit of additional ACGME requirements and standardization of obstetric training in EM.

Our survey findings support the fact that EM residency training may benefit from educational interventions to increase exposure to obstetric patients. At our institution, based on feedback regarding our Ob/Gyn experience, a Longitudinal Ob/Gyn Clinical Experience has been instituted giving second through fourth year residents additional obstetrics experience. Literature has demonstrated that students are generally more satisfied and may learn and retain both knowledge and clinical skills better when trained in a longitudinal curriculum (8, 9). Further, satisfaction surveys have demonstrated that learners prefer this method of training to traditional programs with discrete experiences (8–10).

Other possible interventions to improve resident attitude and knowledge include increasing the time spent on the dedicated Ob/Gyn rotation during the intern year or revisiting the Ob/Gyn experience again during PGYs 3 or 4. Many residency programs are limited by available time for additional clinical responsibilities. In these instances, implementation of structured didactics, web-based learning tools, or simulation-based curricula specifically designed to target resident exposure to obstetric emergencies may ensure a more comprehensive educational experience.

## Limitations

Our study has several limitations. Although adapted from an established study tool and piloted for face validity, our survey did not undergo further validity and reliability testing. The low response rate creates the possibility of participation bias and restricts the generalizability of our findings. However, our study sample did include diversity in terms of PGY levels, regional distribution, and representation from a broad range of US residency programs.

## Conclusions

The identification and management of obstetric emergencies is the cornerstone of EM training. In this exploratory needs assessment of EM residency programs, we found preliminary evidence of a concerning lack of resident comfort regarding obstetric conditions and profound knowledge deficits on core obstetrics topics. These findings should be further investigated in a larger sample of EM residents. Such work may ultimately support the need for a standardized curriculum or other educational interventions that increase exposure to these clinical topics.
